# Clustering the cortical laminae: in vivo parcellation

**DOI:** 10.1007/s00429-023-02748-2

**Published:** 2024-01-09

**Authors:** Ittai Shamir, Yaniv Assaf, Ron Shamir

**Affiliations:** 1 Department of Neurobiology, Faculty of Life Sciences, Tel Aviv University, Tel Aviv, Israel; 2 Sagol School of Neuroscience, Tel Aviv University, Tel Aviv, Israel; 3 Blavatnik School of Computer Science, Tel Aviv University, Tel Aviv, Israel

**Keywords:** Neuroimaging, Cortical layers, Anatomical mapping, Clustering, Algorithms, Cytoarchitecture

## Abstract

The laminar microstructure of the cerebral cortex has distinct anatomical characteristics of the development, function, connectivity, and even various pathologies of the brain. In recent years, multiple neuroimaging studies have utilized magnetic resonance imaging (MRI) relaxometry to visualize and explore this intricate microstructure, successfully delineating the cortical laminar components. Despite this progress, T1 is still primarily considered a direct measure of myeloarchitecture (myelin content), rather than a probe of tissue cytoarchitecture (cellular composition). This study aims to offer a robust, whole-brain validation of T1 imaging as a practical and effective tool for exploring the laminar composition of the cortex. To do so, we cluster complex microstructural cortical datasets of both human (*N* = 30) and macaque (*N* = 1) brains using an adaptation of an algorithm for clustering cell omics profiles. The resulting cluster patterns are then compared to established atlases of cytoarchitectonic features, exhibiting significant correspondence in both species. Lastly, we demonstrate the expanded applicability of T1 imaging by exploring some of the cytoarchitectonic features behind various unique skillsets, such as musicality and athleticism.

## Introduction

### Progress in MRI imaging of T1 layers

The intricate laminar structure of the cerebral cortex was first discovered toward the end of the nineteenth century using ex vivo histological methods, sparking over a century of studies into its assumed roles in the development, function, connectivity, and even pathologies of the brain (Meynert 1872; Bevan Lewis 1879; Ramón y Cajal et al. 1988; García-Cabezas et al. 2019). With the advent of MRI neuroimaging, the cerebral cortex was successfully segmented, delineating its cortical surfaces bordering with underlying white matter and the surrounding cerebrospinal fluid (Fischl 2012). However, the laminar substructure of the cerebral cortex was initially assumed to be beyond the imaging capabilities of MRI. Since then, an increasing number of neuroimaging studies have proposed a variety of MRI imaging modalities and approaches for exploring the laminar composition of the cortex, with T1 relaxometry proving to be one of the most suitable and accurate approaches so far (Clark et al. 1992; Barbier et al. 2002; Bridge and Clare 2006; Duyn et al. 2007; Deistung et al. 2013; Glasser et al. 2014; Lutti et al. 2014; Shafee et al. 2015; Assaf 2019; Van Essen et al. 2019).

The applicability of T1 relaxometry in exploring the cortical laminar composition was established in a series of studies. In 2012, a study characterized the cortical layers in the brains of both humans and rats and compared the resulting T1 clusters to histological findings from the rat brain (Barazany and Assaf 2012). In 2018, a larger scale study used the same inversion recovery (IR) MRI protocol to explore the laminar composition of both humans and rats, concluding that low-resolution T1 mapping is the most appropriate approach for delineating the layers (Lifshits et al. 2018). The study concluded that due to their small physical thickness, the layers are better delineated using high resolution in the T1 relaxation domain, rather than high resolution in the spatial domain. While this conclusion is counterintuitive, it is analogous to diffusion MRI, in which micron-level resolution is achieved not by generating an image at the micron scale, but rather by characterizing a micron-scale phenomenon (Lifshits et al. 2018). In 2019, a complete automated framework was formed for analyzing the cortical laminar composition using low-resolution multi-T1 mapping and a simple surface-based volumetric sampling system (Shamir et al. 2019). What followed were several studies using multi-T1 imaging to explore the role of the cortical laminar composition in different pathologies, such as epilepsy (Lotan et al. 2021), as well as in healthy aging (Tomer et al. 2022). Other studies modeled and explored patterns of cortical connectivity on the laminar level (Shamir and Assaf 2021a, b; Shamir et al. 2022).

The abovementioned studies provide further substantiation for cortical laminar composition analysis using low-resolution, multi-T1 imaging. However, this methodology has so far been limited by the fact that T1 is still not considered a direct measure of cytoarchitecture (cellular composition). This study aims to offer a robust, whole-brain validation of T1 imaging as a practical and effective probe for exploring cortical microstructure on the laminar level.

### Challenges in clustering multilayered surface-based data

**Fig. 1:**
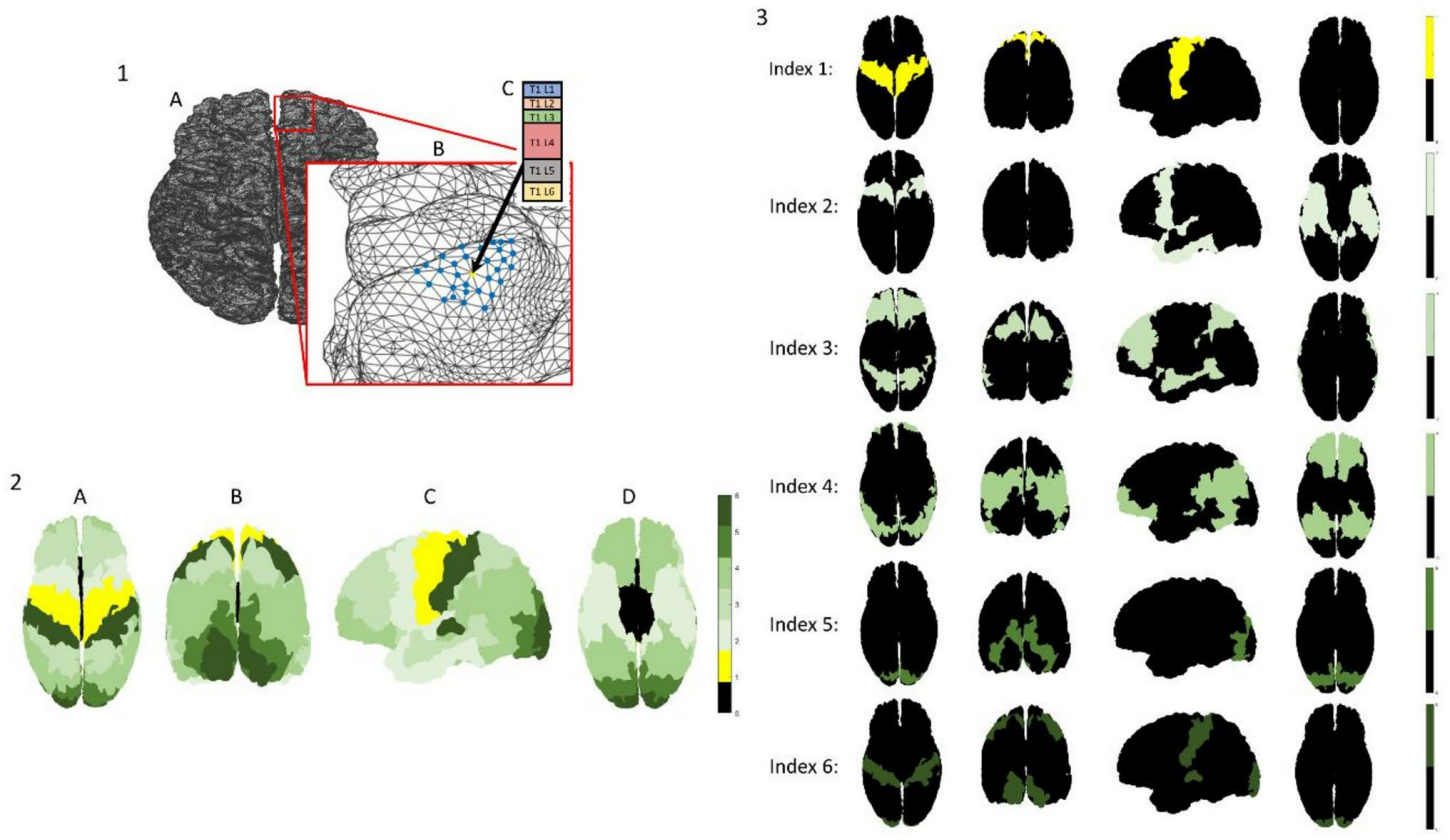
**Multilayered surface-based dataset and a cytoarchitectonic granularity atlas:** (**1**) Multilayered cytoarchitectonic surface-based dataset: the cerebral cortex is represented by a Delaunay triangulation, delineating the mid cortical surfaces of each hemisphere (seen from a superior view in *A*). A single vertex is seen (*yellow*) on a sulcus in the frontal lobe of the right hemisphere, surrounded by its thirty closest neighboring vertices (*blue*) (*B*). The cytoarchitectonic laminar composition at the location of the chosen vertex is shown, including widths of six laminar components: T1 layers 1–6 (colored individually in *C*). (2–3) A cytoarchitectonic atlas of granularity indices: cytoarchitectonic labeling of cortical regions according to granularity indices across the cerebral cortex. Granularity indices: (0) non-neocortical regions, (1) agranular, (2) slightly granular, (3, 4, 5) increasing levels of granule cell presence (3 < 4 < 5), (6) granular cortex. The entire atlas (2) and its components **(3)** can be seen from different viewpoints: (*A*) superior, (*B*) caudal, (*C*) lateral (*left*), (*D*) inferior

Use of the framework for cortical laminar composition analysis (Shamir et al. 2019, 2022; Shamir and Assaf 2021a, b) results in a multilayered, surface-based dataset representing the regionally varying laminar composition across the cortical surfaces of both hemispheres. Despite the established correspondence between T1 layers and the actual cortical layers (Clark et al. 1992; Barbier et al. 2002; Bridge and Clare 2006; Duyn et al. 2007; Barazany and Assaf 2012; Deistung et al. 2013; Glasser et al. 2014; Lutti et al. 2014; Shafee et al. 2015; Lifshits et al. 2018; Assaf 2019; Shamir et al. 2019, 2022; Van Essen et al. 2019; Lotan et al. 2021; Shamir and Assaf 2021a, b; Tomer et al. 2022), T1 is still thought to be more of a direct measure of myeloarchitecture (myelin content) than of cytoarchitecture (Glasser et al. 2016; Shamir and Assaf 2023). This dataset is both multidimensional and geometrically complex: up to six cortical laminar components, representing the regionally varying microstructure of the cortex, are measured across vertices of a Delaunay triangulation, delineating the intricate geometry of the cortex. It is noted that some components (or laminar components) can have the value zero at some vertices. More specifically, for each hemisphere the triangulation consists of ~150,000 vertices, connected by ~300,000 faces, with laminar composition values available for each vertex, including six laminar components corresponding to the widths of T1 layers 1–6 (see Fig. 1, part 1).

We hypothesize that suitable clustering of the T1 layer composition across the entire cortex should correlate to spatially defined cortical regions with distinct cytoarchitectonic features. While many cortical atlases exist, one of the most robust reference atlases for cortical cytoarchitecture is the granularity atlas (von Economo 2009). The granularity atlas divides each hemisphere into about a dozen continuous regions, each labeled with a granularity index of 1–6 according to the level of overall cross-section cellular granularity observed histologically (see Fig. 1, parts 2–3). Its robustness and applicability for our needs lie in the fact that it divides the cortex into regions based on cytoarchitectonic features alone. Since its publication, the granularity atlas has been further discussed (Beul and Hilgetag 2014), made publicly available (Scholtens et al. 2016), and repeatedly utilized (Shamir and Assaf 2021a, b; Parkes et al. 2022; Sajad et al. 2022; Shamir et al. 2022; Wallace et al. 2022; Katsumi et al. 2023).

Analyzing patterns in this complex laminar dataset is no simple task, since the ability to visualize the entire dataset simultaneously is limited, and therefore, accurate whole-brain clustering must be accomplished. The first challenge in clustering the dataset relates to its surface-based nature, in which the spatial locations of the data are not given across pixels or voxels, but rather across vertices on a triangulation surface. The second challenge relates to the dimensionality and low variability of the data, which includes six T1 layer widths with an overall average cortical cross section of approximately two millimeters. The third and final challenge relates to both spatial dispersion and multidimensionality: accurate clustering must take into consideration not only the T1 layer composition at each given data point but also the compositions of its neighboring data points. The importance of identifying neighborhoods of data points with similar laminar compositions (clusters) lies in the expected correspondence with well-defined cortical regions with distinct cytoarchitectonic features. Furthermore, at times even smaller regions vary in their cytoarchitectonic features. For example, gyral caps (peaks) and sulcal fundi (valleys) vary in their overall thickness as well as in their laminar substructure.

### Existing methods for clustering multidimensional, graph-based datasets

One popular approach for clustering data with spatial information is by using graphs. In this approach, graph vertices represent data points in space and graph edges connect vertices that are close in space. Edges can be weighted reflecting the distance between the points. Over the years, a plethora of clustering algorithms have been developed for the problem, including some of the following: similarity graph connectivity clustering (Hartuv and Shamir 2000), density-based clustering using both attribute similarity and spatial proximity (Liu et al. 2012), distributed K-means clustering of mesh networks (Ramesh 2015), and community detection in networks (Girvan and Newman 2002; Traag et al. 2019).

In medical imaging, various algorithmic approaches have been proposed for dealing with graph-based image segmentation (Chu et al. 2002) and density-based image segmentation using super-pixels (Zhang et al. 2017). In the field of MRI neuroimaging, different algorithmic approaches have been developed for threshold-free, surface-based clustering (Lett et al. 2017), as well as for community detection in functional networks (Akiki and Abdallah 2019). Recently, a novel algorithm in the field of omics, which includes genomics, transcriptomics, proteomics, and metabolomics, has been developed for clustering cell omics profiles using the spatial organization of the cells (Singhal et al. 2022). The algorithm, Building Aggregates with a Neighborhood Kernel and Spatial Yardstick (BANKSY), clusters multidimensional omics data across a surface-based spatial representation.

## Methods and materials

### Histological dataset-BigBrain segmentation

The histological dataset was provided by BigBrain, a high-resolution, three-dimensional histological model of the human brain (Amunts et al. 2013). Based on reconstruction of 7,404 histological sections, BigBrain provides a cellular-level resolution of 20 µm of the brain. This dataset was utilized to provide the first whole-brain three-dimensional segmentation of all cortical and laminar surfaces in the human cerebral cortex. The layers were automatically segmented using a convolutional neural network based on histological intensities along cortical profiles sampled between the pial and white matters throughout the cortex. These surfaces were used to evaluate cortical thickness gradients and the contributions of different cortical laminae to these gradients (Wagstyl et al. 2020). Since its publication, this dataset has been made publicly available and repeatedly utilized (e.g., Bazinet et al. 2023; Han et al. 2023; Shafiei et al. 2023). The BigBrain dataset is provided in a surface-based format, including cortical surfaces delineating the borders of all cortical laminar components, from which laminar widths are easily extracted. Data are freely available at: https://bigbrainproject.org/

### Neuroimaging datasets-MRI T1 layers

#### Human (*N* = 33)

The human neuroimaging dataset includes (*N* = 33) healthy human subjects, 16 male and 17 female, 18–78 years old, all neurologically and radiologically healthy with no history of neurological diseases. Of the (*N* = 33) subjects, (*N* = 30) subjects were chosen from the same dataset used by (Shamir et al. 2022) for assessing the overall resulting clustering patterns. The additional (*N* = 3) subjects included were chosen from three groups of interest: a professional athlete, a professional musician, and a multilingual subject (polyglot). These exemplary subjects were chosen for assessing subject-specific features in comparison to the thirty-subject group average. Data for all subjects (*N* = 33 in total) were collected and processed in the same way.

All subjects gave fully informed consent before enrollment in this study. The whole study and the imaging protocols were approved by the institutional review boards of Sheba Medical Center and Tel Aviv University, where the MRI investigations were performed. All methods were performed in accordance with the relevant guidelines and regulations. Each subject was then scanned in a 3 T Magnetom Siemens Prisma (Siemens, Erlangen, Germany) scanner with a 64-channel RF coil and gradient strength of up to 80 mT/m at 200 m/T/s. The scans include the following sequences:

1. A T1-weighted MPRAGE sequence, with the following parameters: TR/TE = 1750/2.6 ms, TI = 900 ms, 1 × 1 × 1 ${{\text{mm}}}^{3}$, 224 × 224 × 160 voxels. Acquisition time was approximately 3.5 min.
2. An inversion recovery echo planar imaging (IR EPI) sequence, with the following parameters: TR/TE = 10,000/30 ms and 60 inversion times spread between 50 ms up to 3000 ms, 3 × 3 × 3 ${{\text{mm}}}^{3}$, 68 × 68 × 42 voxels, each voxel fitted with up to 7 discrete and weighted T1 values using seven individual exponential fits, based on an assumption regarding the number of T1 components in the tissue (Lifshits et al. 2018). Acquisition time was approximately 12 min.

Total scan time for both sequences was approximately 15.5 min.

**Fig. 2:**
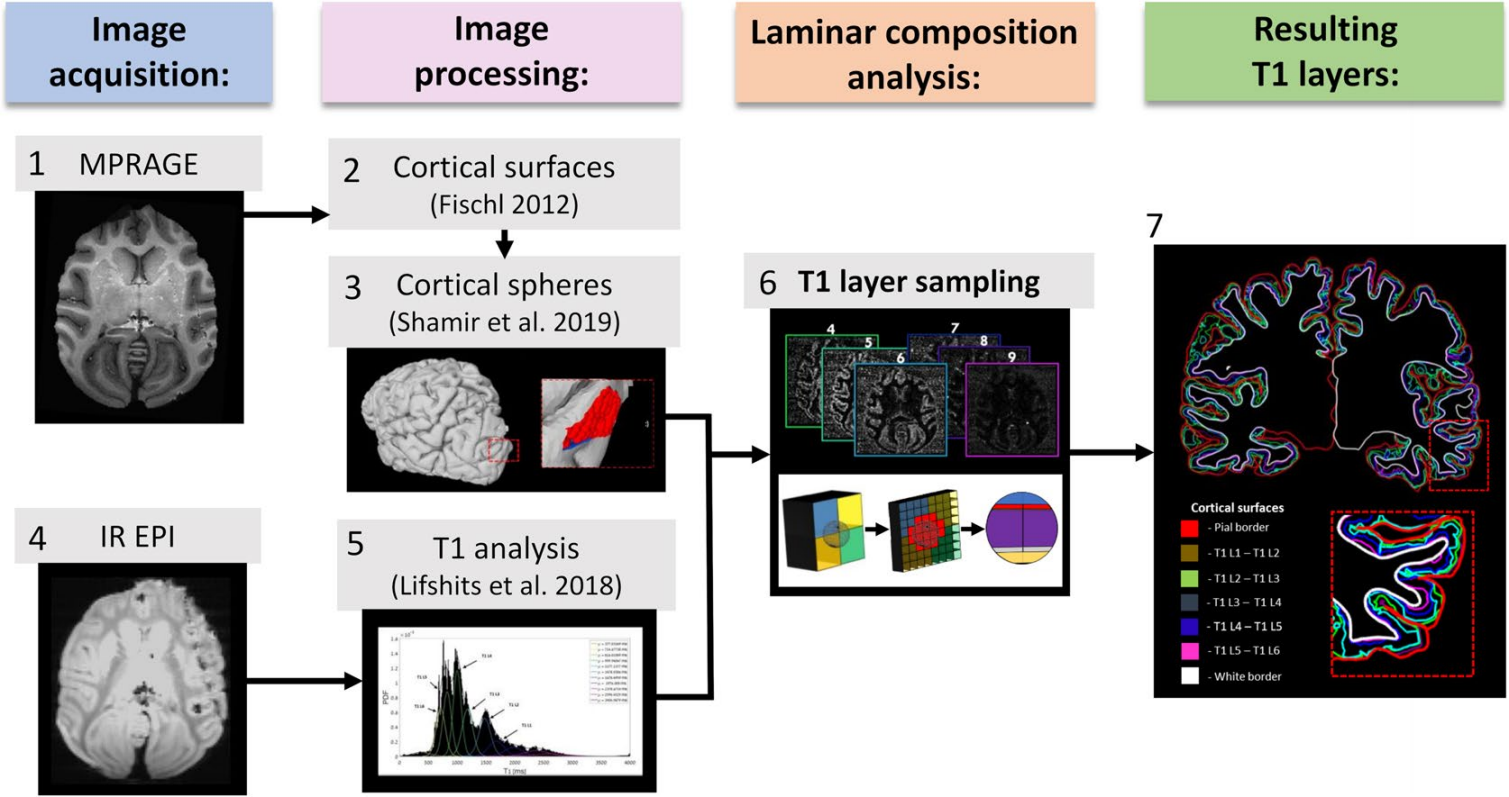
**An outline of the framework for analyzing the cortical laminar composition:** (*1*) MPRAGE image acquisition (*axial*) as an anatomical reference of the overcall cortical geometry. (*2*) Cortical surfaces extraction, including the inner and outer cortical borders. (*3*) Cortical spheres formation, including a system of volumetric spheres across the cortex. (*4*) The outer cortical surface of the left hemisphere (*gray*) and the cortical spheres beneath it (*red*) are seen from a caudal viewpoint. (*5*) IR EPI image acquisition (axial) for characterizing composition of the cortical layers. (*6*) T1 analysis using probabilistic classification of the T1 histogram (*black bins*) into a mixture of t-distributions (*colored lines*) corresponding to different brain tissues. (*7*) T1 layer sampling of t-distributions 2–9, corresponding to T1 layers VI-I (*top*), from a weighted voxel-wise basis to the system of cortical spheres (*bottom*). (*8*) Resulting T1 layers 1–6, represented here by the cortical surfaces bordering with each component (coronal view of both hemispheres). The thirty-subject average dataset, including both standard connectivity and laminar connectivity, is freely available at https://github.com/ittais/Laminar_Connectivity

The first sequence was used as an anatomical reference, as well as for delineating the cortical surfaces, and the second sequence was used for characterizing the cortical layers. The acquired images were processed according to the framework for cortical laminar composition analysis. In general terms, the framework starts with estimation of multiple T1 components per voxel in the IR EPI images using an IR decay function fit. The whole-brain histogram of weighted T1 values then undergoes probabilistic classification into different brain tissues using a t-distribution mixture model. After precise image registration, the cortical surfaces are extracted from the MPRAGE image and a cortical sampling system of virtual spheres is built within the delineated cortical volume. The per-voxel classified T1 values are then sampled within the cortical spheres using a super-resolution solution. The result is an estimation of T1 layer values across the cortical surfaces (Shamir et al. 2019; Shamir and Assaf 2021b; Shamir et al. 2022; Shamir and Assaf 2023, see Fig. 2).

#### Macaque (*N* = 1):

The macaque neuroimaging dataset is taken from Shamir and Assaf (2021b) and includes a single excised macaque brain that was obtained from the Mammalian MRI (MaMI) database (Assaf et al. 2020). No animals were deliberately euthanized for the present study. The excised macaque brain was formalin fixated, and some 24 h before MRI it was placed in phosphate-buffered saline for rehydration. For the scan, the brain was placed in a plastic bag and immersed in fluorinated oil (Flourinert, 3 M) to minimize image artifacts caused by magnetic susceptibility effects. The brain was scanned on a 7 T/30 Bruker scanner with a 660 mT/m gradient system. The high-resolution scans include the following sequences:

1. A T1w sequence with a 3D modified driven equilibrium Fourier transform (MDEFT), with the following parameters: TR/TE = 1300/2.9 ms, TI = 400 ms, 0.2 × 0.2 × 0.2 ${{\text{mm}}}^{3}$, 300 × 360 × 220 voxels. Acquisition time was approximately 2 h and 13 min.
2. An inversion recovery sequence using 3D FLASH, with the following parameters: TR/TE = 1300/4.672 ms and 44 inversion times spread between 25 ms up to 1000 ms, voxel size 0.67 × 0.67 × 0.67 ${{\text{mm}}}^{3}$, 96 × 96 × 68 voxels, each voxel fitted with up to 8 discrete and weighted T1 values using eight individual exponential fits (similarly to (Lifshits et al. 2018)). Acquisition time was approximately 69 h and 34 min.

Total scan time for both sequences was approximately 71 h and 47 min.

The first sequence was used as an anatomical reference with high gray/white matter contrast for segmentation and estimation of cortical surfaces (like the clinical MPRAGE sequence), and the second sequence was used for characterizing the cortical layers. The acquired images were processed according to the framework for cortical laminar composition analysis, using an adaptation for the macaque dataset (Shamir et al. 2019, 2022; Shamir and Assaf 2021b).

### Ground truth reference-granularity atlas

In performance analysis of algorithms, to evaluate the quality of a solution, it is compared to an independently available, established reference resource, defined—for the sake of the evaluation—as the ideal expected result, or the “ground truth”. Here, the cortical atlas of granularity indices (as shown in Fig. 1, parts 2–3) was used as the ground truth reference for cytoarchitecture. The granularity atlas labels cortical regions according to the overall level of cytoarchitectonic granularity observed histologically across cortical cross sections, from agranular low order cortex to high order granular cortex. The atlas was generated by manual labeling of the 44 cytoarchitectonic regions (per hemisphere) of the von Economo–Koskinas atlas, resulting in about a dozen continuous regions with varying degrees of granularity (von Economo 2009; Beul and Hilgetag 2014; Scholtens et al. 2016; Shamir and Assaf 2021a). A similar atlas of cortical granularity was used for the macaque brain, based on a map of cytoarchitectonic features across the primate cortex (Beul and Hilgetag 2019; Shamir and Assaf 2021b).

### BANKSY algorithm adaptation

In this study we use the BANKSY algorithm for spatial clustering (Singhal et al. 2022). The algorithm was originally developed for clustering cells into types based on their transcription profiles and spatial organization. We adapt and implement the algorithm on surface-based cortical laminar composition datasets, both histological (BigBrain) and neuroimaging (MRI T1 layers). Our implementation of the algorithm includes the following steps:

1. Neighbor-augmented matrix construction:
  For each hemisphere, we construct a neighbor-augmented matrix as described by Singhal et al. (2022) using cortical layer width values instead of genomic transcription expression values. The matrix B is m $\times$ n, where n $\cong$ 150,000 columns, corresponding to vertices on the cortical surface, and m = 12 (see Eq. 1). Each column in the top half of the matrix ${B}_{{\text{top}}}$ includes the six cortical layer widths for the vertex, and the bottom half ${B}_{{\text{bottom}}}$ includes the six layer widths averaged over the neighborhood of the vertex. The contribution of each component is controlled by parameter $\lambda$ (see Eqs. 1–3). The neighborhood values are calculated by locating the thirty nearest vertices on the cortical surface and averaging them using the inverse of the distance to the original vertex, after normalizing the sum of the distances to one (see Eqs. 4 and 5).
  1 $$B = \left[ {\begin{array}{*{20}c} {\sqrt {1 - \lambda } \cdot } & {B_{{{\text{top}}}} } \\ {\lambda \cdot } & {B_{{{\text{bottom}}}} } \\ \end{array} } \right]$$
  where $\lambda$ is the neighborhood weight, we used $\lambda =0.3$ (Singhal et al. 2022).
  2 $$B_{{{\text{top}}}} = [C_{1} \,C_{2} \, \ldots \,C_{n} ]$$
  where ${C}_{{\text{i}}}$ is the cortical layer widths for vertex *i*.
  3 $$B_{{{\text{bottom}}}} = [\gamma_{1} \,\gamma_{2} \, \ldots \gamma_{n} ]$$
  where ${\gamma }_{{\text{i}}}$ is the average cortical layer widths of neighborhood of vertex *i*.
  4 $$\gamma_{i} = \sum\nolimits_{j = 1}^{30} {w_{ij} \cdot C_{j} }$$
  where ${C}_{{\text{j}}}$ is the cortical layer widths for neighbor *j* of vertex i, ${w}_{ij}$—weight for neighbor *j*.
  5 $$w_{ij} = \frac{{\frac{1}{{r_{ij} }}}}{{\sum\nolimits_{q = 1}^{30} {\frac{1}{{r_{iq} }}} }}$$
  where ${r}_{ij}$ is the distance of neighbor *j* from vertex *i*.
  For the histological dataset, because of its assumed high resolution and accuracy, we only used the six layer width values for each vertex. In other words, the neighbor-augmented matrix was ~150,000 $\times$ 12 as described above.
  For the neuroimaging datasets, because the T1 layers correspond to laminar components and lack a one-to-one correspondence to the histological cortical layers, we used the six layer width values as well as the overall cortical width values. The addition of the overall cortical width can be further explained by the relatively more established measurement of the cortical cross-section segmentation using MRI (Fischl 2012). Overall, the neighbor-augmented matrix was ~150,000 $\times$ 14, including six cortical layer widths and the overall cortical width of the vertex, and six averaged cortical layer widths and the averaged overall cortical width of its neighborhood.
2. Neighbor-augmented matrix clustering:
  The neighbor-augmented matrices for both hemispheres are concatenated into a single whole-brain matrix for clustering (see Eq. 6).
  6 $$B_{{{\text{whole}}\,{\text{brain}}}} = [B_{{{\text{left}}\,{\text{hemisphere}}}} \,B_{{{\text{right}}\,{\text{hemisphere}}}} ].$$
  For both the histological and the neuroimaging datasets we clustered the corresponding matrices using a simple unsupervised K-means clustering algorithm, which partitions the vectors (~150,000 × 2 matrix columns) into K clusters. Aside from the variation in the construction of the neighbor-augmented vectors (see step 1), we used a different number of clusters K for the histological and neuroimaging datasets. For each dataset, K was selected according to visual assessment using global matching of regional patterns to those in the granularity atlas. For the histological dataset we got the best results when using K = 6, and for the neuroimaging datasets (N = 30 subjects) we repeatedly got the best results using K = 4. For the first neuroimaging subject, the clusters were relabeled 1–4 in increasing levels of average granularity according to the granularity atlas (see Fig. 1, parts 2–3). To obtain consistent cluster labeling across subjects, the K-means solution of one subject was used as seed for all other (N = 29) subjects. For the macaque neuroimaging dataset, we got the best results when using K = 5.
3. Evaluating the clustering results:
  After assigning a label to each vertex, the clusters were plotted across the cortical surfaces and visually assessed for hemispheric symmetry and overall similarity to the granularity atlas using global matching of regional patterns. To integrate the results across subjects, we used the Brainnetome atlas, which partitions the cortex into 210 cortical regions, 105 regions per hemisphere (Fan et al. 2016). Three visual and quantitative assessments were then performed:
  - Hemispheric symmetry:
    i. A cross-subject cluster label was assigned to each cortical region according to a majority vote of all vertices in that region across all subjects (N = 30). To visually assess the symmetry, the clusters were plotted across the cortical surfaces.
    ii. To quantitatively assess the level of symmetry, for each subject, each region was assigned a per-subject label according to majority vote of its vertex labels. To measure symmetry, we used the pairing of regions in the left and right hemispheres. Symmetry for a single subject was defined as the percentage of pairs of regions that had the same label. Cohort symmetry was defined in the same way using the cross-subject cluster labels (see a(i)). Symmetry for randomly spatially permuted datasets was computed for comparison.
  - Inter-subject variability:
    To assess inter-subject variability, the per-subject region labels described in a(ii) were used. The standard deviation in cluster assignment per region across all (*N* = 30) subjects was then measured and plotted across the cortical surfaces.
  - Similarity to granularity atlas:
    To visually assess the similarity, the cross-subject region labels described in a(i) were used. The clusters were plotted across the cortical surfaces and compared to a simplified version of the granularity atlas. To quantitatively assess the correspondence, a hypergeometric test was performed (Fisher 1922, 1992; Liddell 1976). The hypergeometric test, also known as Fisher’s exact test, uses the hypergeometric distribution to measure the statistical significance of drawing a sample consisting of a specific number of at least k successes out of a total of n draws from a given population size containing a given number of successes, assuming that draws are done independently at random without replacement. This test was used for comparing the resulting clusters to the simplified groups of granularity indices. For each index and cluster, the number of regions in that index that belong to that cluster was computed, and the significance of the index-cluster overlap was computed. P-values were corrected using the Bonferroni correction for multiple comparisons.
  - Subject-specific features:
    To visually assess the relative granularity features of the (*N* = 3) exemplary subjects, for each subject clusters were plotted in comparison to the thirty-subject regional majority vote. Regions with clusters corresponding to higher granularity levels than the majority vote are presented in hot colors, and regions with lower granularity level are presented in cold colors.

## Results

The same basic clustering algorithm was used for both the histological and the neuroimaging datasets, including two main parameters: neighborhood size and neighborhood weight. The neighborhood size, or number of vertices nearest to a given vertex to be averaged, was chosen by testing multiple values ranging between 0 and 100. When testing both types of datasets and increasing the neighborhood size incrementally, results did not change much beyond a size of thirty neighboring vertices. Accordingly, a neighborhood size of thirty vertices was deemed satisfactory for both types of datasets. The second parameter is the neighborhood weight ($\lambda$), which weighs the relative contributions of the two components per vertex: its individual laminar composition value, and the averaged values of its neighborhood. The neighborhood weight was chosen by testing multiple values ranging between 0, representing no neighborhood contribution, and 1, representing solely neighborhood contribution. For both types of datasets, $\lambda =0.3$ resulted in the most visually distinct and cohesive clusters across the cortex and was consequently deemed most fitting. It is worth noting that the same neighborhood weight was used for omics data in the study that introduced BANKSY (Singhal et al. 2022).

### Clustering the histological dataset

**Fig. 3:**
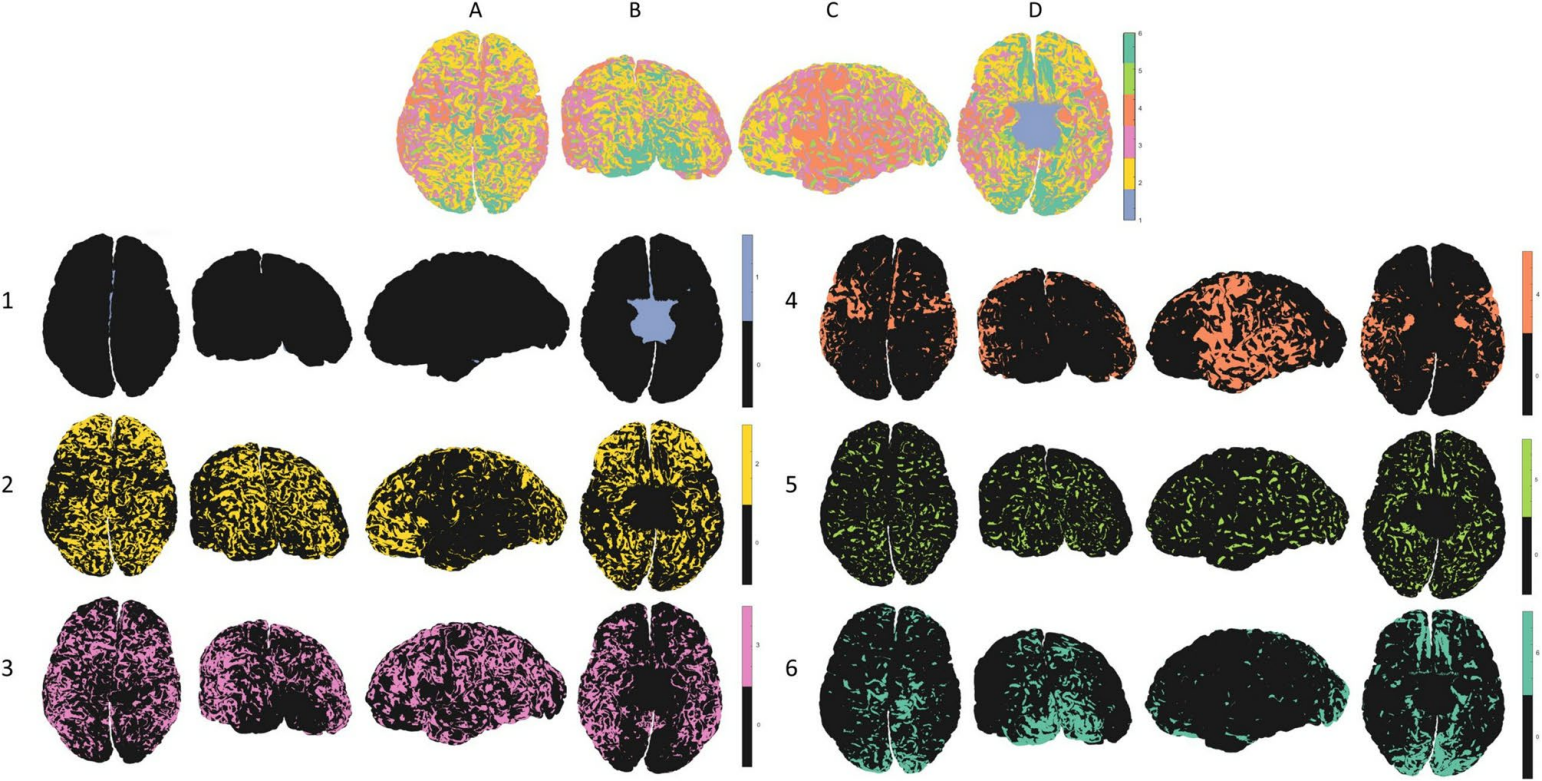
**Clustering histological dataset (BigBrain):** All six clusters can be seen in the top row, and each individual cluster can be seen on the rows below (1–6): (*1*) non-neocortical regions, (*2*, *3*, *4*) agranular to increasingly granular cortices, (*5*, *6*) increasingly granular to granular cortex. The clusters can be seen from different viewpoints: (*A*) superior, (*B*) caudal, (*C*) lateral (*left*), (*D*) inferior

K-means clustering of the neighbor-augmented matrix for the histological dataset resulted in six distinct clusters that show correspondence to most of the six granularity indices in the granularity atlas (see results in Fig. 3). When examining the resulting six clusters, global matching of regional patterns appears. Firstly, overall visual assessment of the clusters shows high hemispheric symmetry between the left and right hemispheres. Secondly, the methodology successfully delineated occipital regions with increasingly high granularity, alongside successful delineation of temporal regions with mid-to-low granularity levels (Fig. 3, top row B and C, respectively). When examining the six clusters individually, additional interesting features appear. As seen, the process successfully delineated non-neocortical regions (Fig. 3, row 1). This result is expected given the fact that no layer data are included for non-neocortical regions that have no granularity index labeling. The other clusters seem to correspond to regions with increasing granularity indices, from frontal regions that are considered more agranular (Fig. 3, rows 2 and 3), to temporal and parietal regions that are considered more granular (Fig. 3, rows 4 and 5), up to occipital regions that are considered entirely granular (Fig. 3, row 6). A third notable feature across all individual clusters is the differentiation across the cortical folding, i.e., gyral caps and sulcal fundi, which are known to differ in both thickness and laminar composition (Wagstyl et al. 2020; MacDonald et al. 2000).

The effective clustering of cortical layers in the histological dataset, accurately identifying cortical regions with varying cytoarchitectonic features, provided a proof of concept for the consequent application of the BANKSY clustering algorithm on our T1 layer neuroimaging datasets.

### Clustering the neuroimaging datasets

**Fig. 4:**
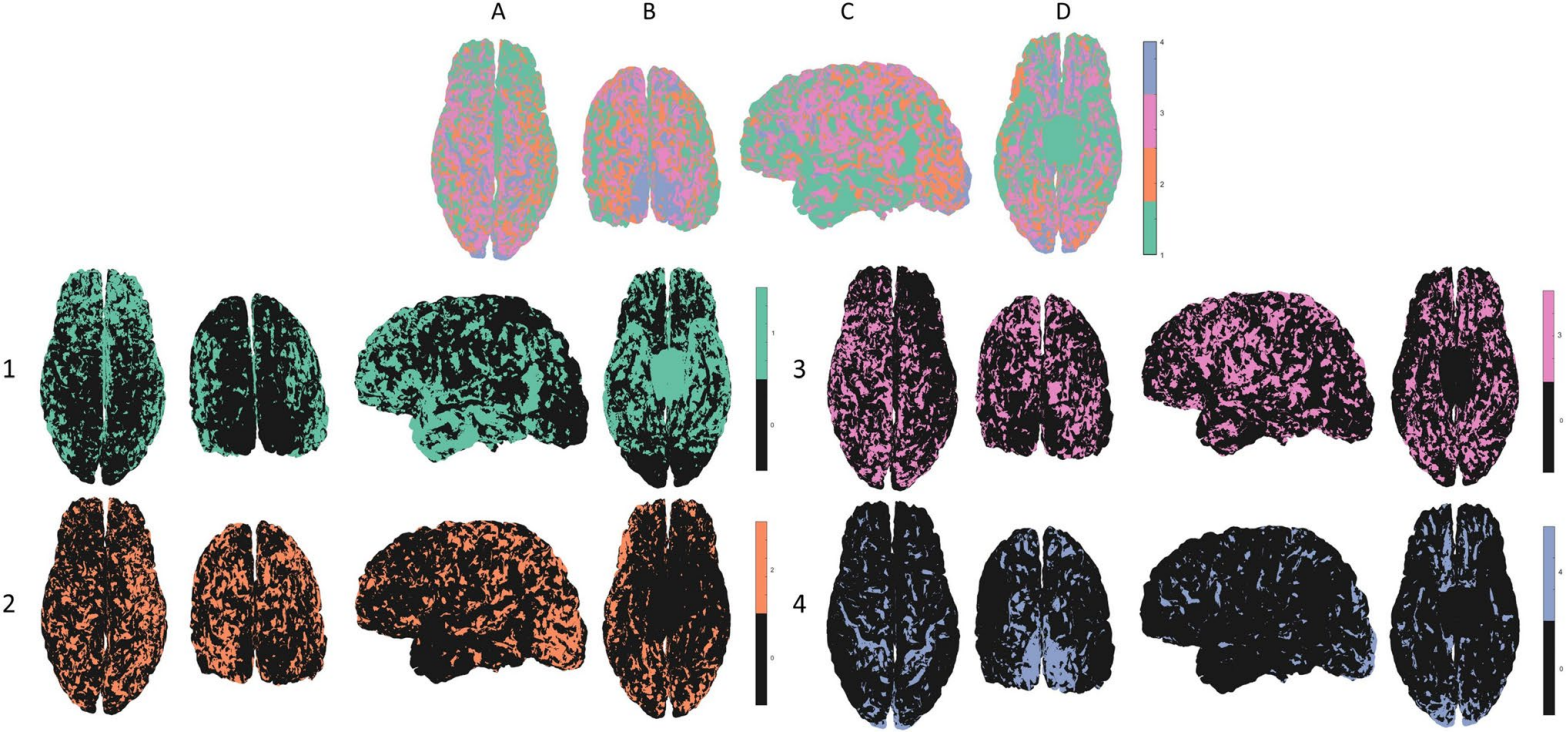
**Clustering neuroimaging dataset for a single subject (MRI T1 layers):** All four clusters can be seen in the top row, and each individual cluster can be seen on the rows below (1–4): (*1*) non-neocortical regions, agranular cortex, (*2*, *3*) increasingly granular, (*4*) granular cortex. The clusters can be seen from different viewpoints: (*A*) superior, (*B*) caudal, (*C*) lateral (*left*), (*D*) inferior

The results of K-means clustering of the neighbor-augmented matrix for the neuroimaging dataset with *K* = 4 clusters show correspondence to a coarser division of the granularity indices in the granularity atlas (see Fig. 4). When examining all four clusters simultaneously, once again it appears that the process successfully delineated occipital regions with increasingly high granularity, alongside successful delineation of temporal regions with mixed granularity levels (Fig. 4, top row B and C, respectively). Additionally, relatively high hemispheric symmetry can be seen from visual assessment of clusters 1 and 4. When examining the four clusters individually, some interesting features appear. As seen, the process delineated non-neocortical regions but merged it with the agranular cortex (Fig. 4, row 1). While the histological dataset only includes laminar information for neocortical regions, the neuroimaging datasets also include information for non-neocortical regions, which are characterized by a laminar composition of only three to four layers. The other clusters seem to correspond to a coarser division into regions with increasing granularity indices, from frontal regions that are considered less granular (Fig. 4, row 2), to temporal and parietal regions that are considered more granular (Fig. 4, row 3), ending once more in occipital regions with high granularity (Fig. 4, row 4).

**Fig. 5:**
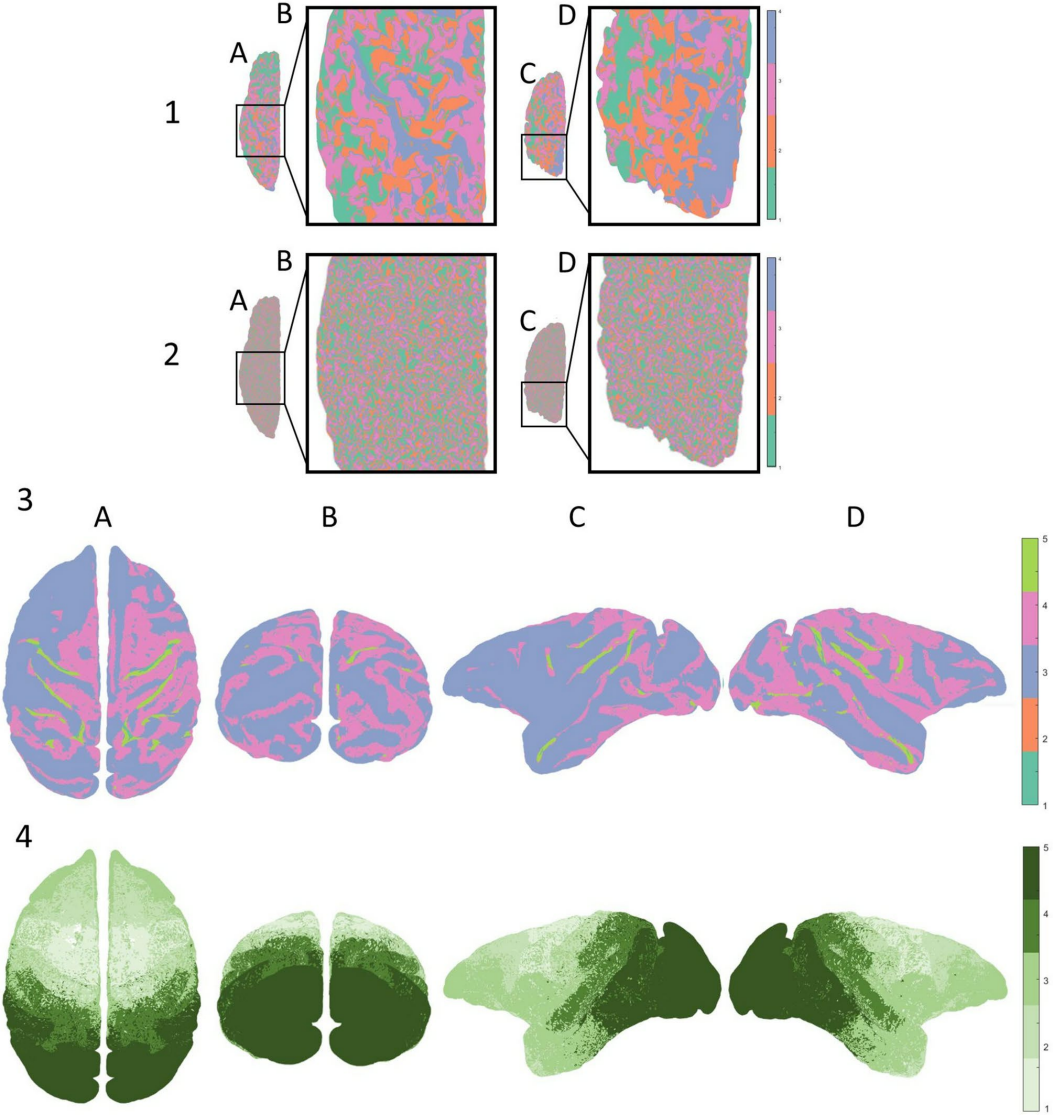
**Clustering different neuroimaging datasets:** Differences in clusters between an original and a randomized neuroimaging dataset (human): (*1*) Original dataset: the resulting clusters in a neuroimaging dataset showcase a differentiation between gyri and sulci across the cortical folding. (*2*) Randomized dataset: the same neuroimaging dataset was permuted spatially and then clustered using the same algorithm, resulting in no delineation of any regions of cytoarchitectonic importance. The results across the left hemisphere can be seen from a superior view (*A*) of the postcentral gyrus (*B*), and from a caudal view (*C*) of the primary visual cortex (*D*). (*3*) Clustering macaque neuroimaging dataset: of the five resulting clusters, four dominant clusters can be seen from different viewpoints: (*A*) superior, (*B*) caudal, (*C*) lateral (*left*), (*D*) lateral (*right*). (*4*) Granularity atlas (reduced): the cytoarchitectonic atlas of granularity indices (as seen in Fig. 1), adapted for the macaque brain (Bridge and Clare 2006; von Economo 2009) and reduced to five components

Similar patterns were observed in the clustering results of all (*N* = 30) subjects. The differentiation between cytoarchitecture in gyri and sulci that was previously observed for the histological dataset is also noticeable here across all individual clusters (see Fig. 5, part 1). Furthermore, when we randomly permute the spatial locations of the neighbor-augmented vectors in the neuroimaging dataset and then apply the same adaptation of the BANKSY algorithm, any delineation of regions of cytoarchitectonic importance disappears (see Fig. 5, part 2).

When examining the six resulting clusters for the excised Macaque brain, a similar cytoarchitectonic differentiation between gyri and sulci appears (see Fig. 5, part 3). While the excised macaque brain displays fewer dominant clusters, presumably relating to formalin fixation, the resulting clusters display high hemispheric symmetry and a clear delineation of regions relating to the macaque motor cortex.

**Fig. 6:**
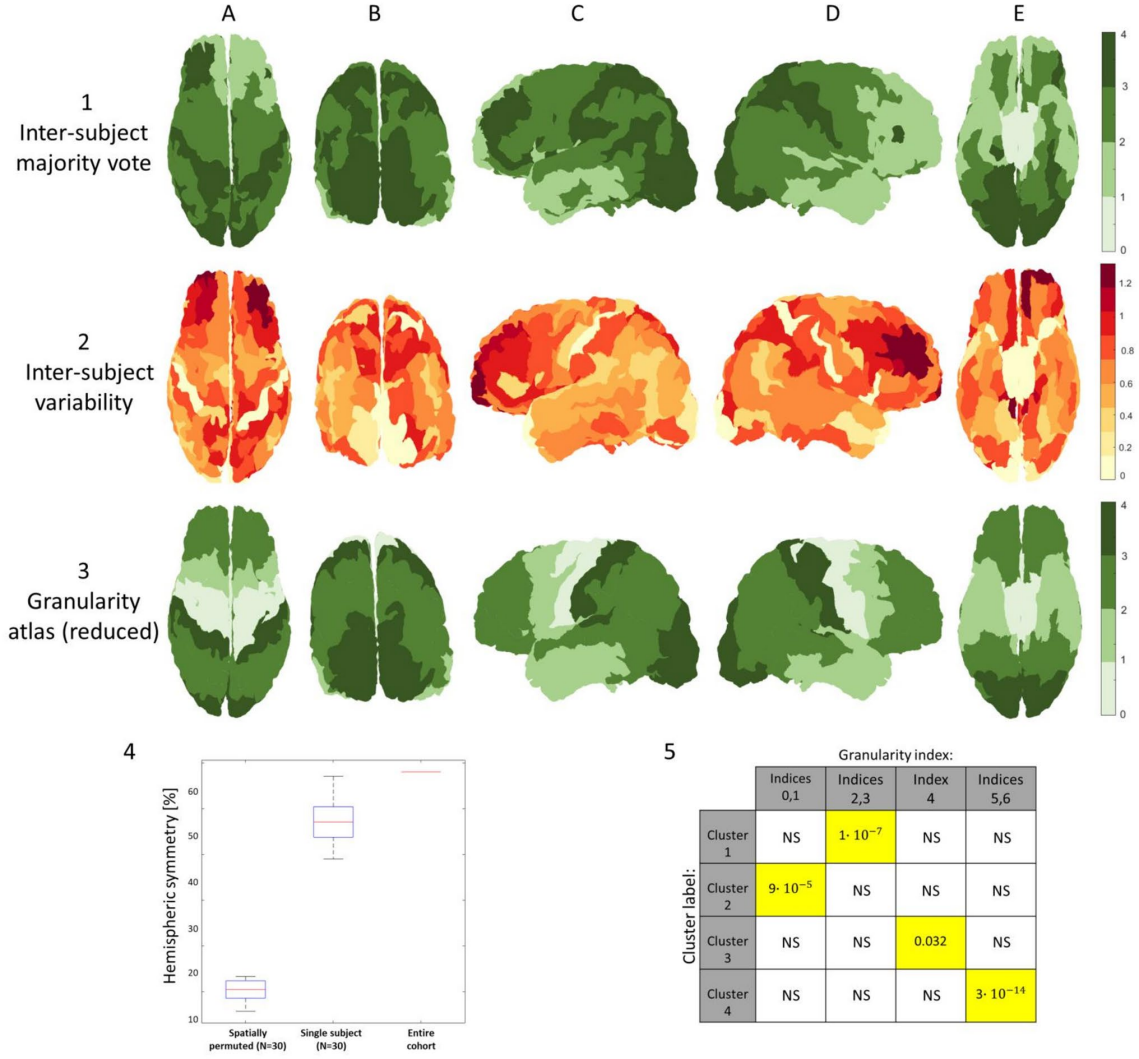
**Inter-subject (*N* = 30) clustering results and quantitative assessments:** (1) Inter-subject majority vote: the most common cluster assigned to vertices belonging to each Brainnetome atlas region, evaluated across all subjects (*N* = 30). (2) Inter-subject variability: the standard deviation of the majority vote clusters across Brainnetome atlas regions, evaluated across all subjects (*N* = 30). (3) Granularity atlas (reduced): the cytoarchitectonic atlas of granularity indices (as seen in Fig. 1), reduced from the original six components to a coarser division including four components. Images are shown from different viewpoints: (*A*) superior, (*B*) caudal, (*C*) lateral (*left*), (*D*) lateral (*right*), (*E*) inferior. (*4*) Hemispheric symmetry: boxplots of the distribution of the fraction of pairs of corresponding cortical regions (left and right hemispheres, including 105 pairs of regions) that match in their cluster assignment. Three distributions are shown: randomly spatially permuted datasets (*N* = 30), per-subject majority vote (*N* = 30), and majority vote for the entire cohort (all subjects). (*5*) Similarity to granularity atlas: hypergeometric *p *values for the correspondence between the four cross-subject clusters and four groups of granularity indices. *P *values were Bonferroni corrected for multiple comparisons. Significantly correlated pairs (*p *value <0.05) are marked in *yellow*

To further evaluate the performance of the algorithm on the neuroimaging datasets, we computed per-subject cluster assignment to each region using majority voting and measured the standard deviation of results across all (*N* = 30) subjects. The regional majority vote was also used for measuring the overall accuracy of the results on a regional level, by finding cross-subject cluster assignment to each region using majority vote of the points in the region across all subjects (see Fig. 6, part 1). Standard deviation was used for measuring variability in per-subject regional cluster assignment across subjects (see Fig. 6, part 2). The evaluation reestablishes the coarser delineation of overall granularity patterns: high granularity in occipital regions and in the postcentral gyrus, mixed granularity in temporal regions, low granularity in frontal regions (merged with non-neocortical regions), and increasing granularity in temporal and parietal regions. When assessing hemispheric symmetry, some asymmetry appears, particularly in regions with mixed granularity indices, such as frontal and temporal regions, which also exhibit high inter-subject variability. To quantitatively assess the hemispheric asymmetry, we measured the percentage of pairs of corresponding regions (mirroring regions between hemispheres, including 105 pairs of regions) that differ in their cluster assignment. The datasets include the following three categories: cross-subject region labels, per-subject region labels (*N* = 30), and per-subject region labels on randomly spatially permuted datasets (*N* = 30). The results show high symmetry values for the original datasets in comparison to the spatially permuted datasets (see Fig. 6, part 4).To quantitatively assess the overall similarity to the granularity atlas (shown in Fig. 6, part 3), we measured hypergeometric scores, testing the correspondence between the four clusters and four groups of granularity indices. Significant correspondence was found between cluster 1 and granularity indices 2–3, cluster 2 and indices 0–1, cluster 3 and index 4, and between cluster 4 and indices 5–6 (see Fig. 6, part 5).

**Fig. 7:**
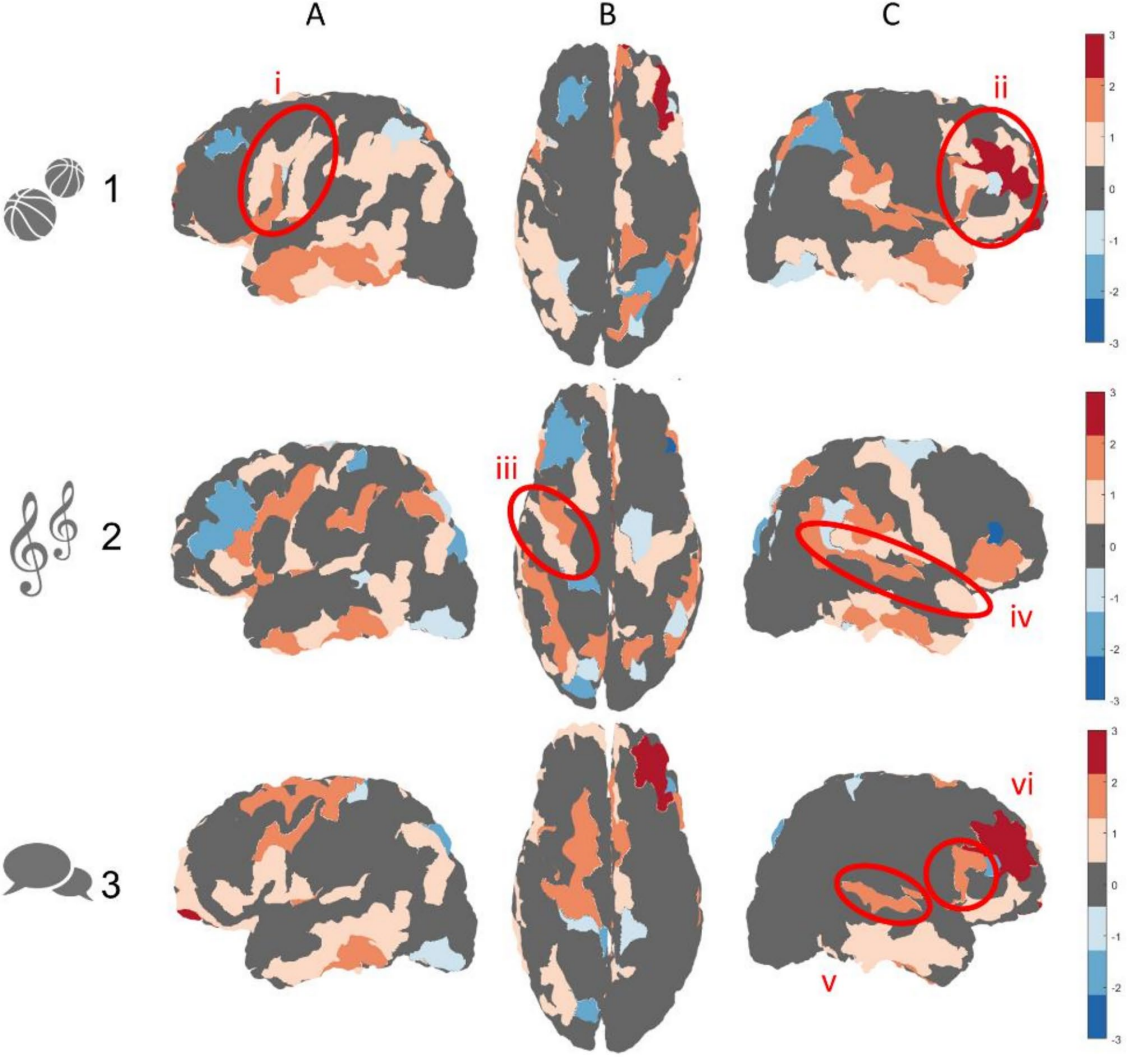
**Relative clustering results for exemplary subjects (*N* = 3) from different groups of interest:** Clustering results are shown in relation to the thirty-subject majority vote (presented in Fig. 6, part 1), where regions with higher granularity levels than the majority vote are presented in hot colors and regions with lower granularity level are presented in cold colors: (*1*) A professional athlete (*N* = 1): relatively higher granularity in motor and premotor regions (*i*), as well as frontal regions (*ii*). (*2*) A professional musician (*N* = 1): relatively higher granularity in motor (*iii*) and auditory (*iv*) regions. (*3*) A polyglot, or person with fluency in multiple languages (*N* = 1): relatively higher granularity in regions associated with language perception and formation (*v* and *vi*). It should be noted that while the professional athlete and musician are both right handed, the polyglot is left handed. Images are shown from different viewpoints: (*A*) lateral (*left*), (*B*) superior, (*C*) lateral (*right*)

To examine subject-specific cytoarchitectonic features, we applied the clustering methodology on each of the (*N* = 3) exemplary subjects, including an athlete, a musician, and a polyglot. The clustering results for these exemplary subjects are shown in relation to the thirty-subject majority vote (no statistical calculation performed here, see Fig. 7). The results show several notable features in concordance with prior neuroanatomical knowledge: the professional athlete exhibits relatively higher granularity in motor and premotor regions, as well as frontal regions (see Fig. 7, part 1), the professional musician exhibits higher granularity in motor and auditory regions (see Fig. 7, part 2), and the polyglot exhibits higher granularity in regions associated with language perception and formation (see Fig. 7, part 3). Subject handedness is also apparent in the results: both the athlete and the musician are right handed, and accordingly they exhibit higher granularity in motor regions of the left hemisphere, while the polyglot is left handed and accordingly exhibits high granularity in mirrored language regions of the right hemisphere.

## Discussion

In this study we cluster microstructural multilayered surface-based data in the cerebral cortex using adaptations of an omics algorithm called Building Aggregates with a Neighborhood Kernel and Spatial Yardstick (BANKSY). This algorithm was developed in the field of omics for clustering genomic and biological datasets based on both cell types and tissue domains and it has been shown to outperform related clustering methods for multiple types of datasets, including successful delineation of the cortical layers by clustering omics data from the dorsolateral prefrontal cortex (Singhal et al. 2022). The same basic clustering methodology was used for both the histological and the neuroimaging datasets. However, its adaptations for these two types of datasets differed in two principal ways: (1) the inclusion, or exclusion, of the overall cortical thickness in the input vector; (2) a varying number of clusters generating.

We first adapted the algorithm for the BigBrain histological dataset (Amunts et al. 2013), a high-resolution three-dimensional segmentation of all cortical and laminar surfaces in the brain (Wagstyl et al. 2020). This initial adaptation involved the use of the six width values of the laminar components and clustering into *K* = 6 clusters, resulting in delineation of multiple cortical regions with distinct cytoarchitectonic features, including non-neocortical regions, regions with increasing granularity from frontal to temporal and parietal regions, and high granularity regions in the occipital cortex as well as the postcentral gyrus. This adaptation provided an initial proof of concept for the applicability of the BANKSY methodology for other surface-based cytoarchitectonic datasets.

We then adapted the algorithm for the neuroimaging datasets, which include the laminar composition of six T1 layers across the cortical surfaces of (*N* = 30) healthy subjects (Shamir et al. 2022). For this adaptation of the algorithm, we achieved optimal results when using the widths of the six laminar components combined with the overall cortical width, all clustered into *K* = 4 clusters. The inclusion of the overall cortical thickness in the input vector for the neuroimaging dataset can be explained due to the relatively lower resolution of the MRI data, as well as the more well-established and well-documented nature of MRI segmentation of overall cortical thickness. The use of (*K* = 4) clusters when clustering the neuroimaging datasets can be explained by the relatively lower resolution of the neuroimaging datasets, compared to the ultrahigh resolution of the BigBrain histological dataset. Once again, the clustering resulted in delineation of multiple regions with distinct cytoarchitectonic features, including regions with increasing granularity from frontal to temporal and parietal regions, and high granularity in occipital regions as well as in the postcentral gyrus.

One of the main limiting factors of this study lies in the relatively small sample size tested, including thirty subjects. However, future studies can use the framework detailed here on a wider scale of subjects. Additional limitations relate to the qualitative nature of the selection process of some algorithm parameters, primarily the number of clusters generated. This process involves visual assessment of the results, which necessitates a level of neuroanatomical expertise. One final limitation involves the use of the granularity atlas as a gold standard reference for cortical cytoarchitecture. While many cytoarchitectonic atlases exist, none provide an actual in vivo, subject-specific reference.

The resulting clusters in both adaptations of the algorithm are characterized by a “patchy” appearance, compared to the more uniform nature of our chosen reference atlas of cytoarchitectonic features (von Economo 2009). The “patchiness” appears to relate to a differentiation between gyri and sulci across the cortical folding, known to vary in both overall thickness as well as in laminar composition (Wagstyl et al. 2020; MacDonald et al. 2000). This explanation is strengthened by the fact that this feature appears in both the histological dataset, which includes only laminar composition as input, as well as in the neuroimaging datasets, which also incorporates the overall cortical thickness as input. The difference in uniformity across cortical regions can also be attributed to methodological differences relating to the labeling process of the granularity atlas. This atlas is the result of a manual labeling process by histologists over a century ago according to cellular features observed across entire cortical regions. By comparison, our clustering methodology involves unsupervised labeling of present-day datasets according to assumed cellular features on a vertex-wise basis. The vertex-wise clustering better suits the nature of the neuroimaging datasets and the regional variability in laminar composition. Nevertheless, to better assess the accuracy of the results quantitatively we used a majority vote for clusters across cortical regions. Our analysis found a significant correspondence between each of the four resulting clusters and a different set of granularity indices. Furthermore, examination of the clustering results for three exemplary subjects with unique skills highlights the applicability of this framework in the exploration of some of the structural mechanisms and cytoarchitectonic features behind different skillsets.

It should be noted that this study does not claim to offer a single “optimal” cortical parcellation based on cytoarchitecture. Multiple other cytoarchitectonic parcellations were proposed, based on other modalities (Glasser et al. 2016; Palomero-Gallagher and Zilles 2019). Our study offers an effective solution to the challenge of clustering multilayered, surface-based datasets that represent the regionally varying laminar composition across the cortex. By doing so, three main goals are achieved. First and foremost, the results highlight the role of MRI neuroimaging as a probe of tissue cytoarchitecture, by providing a validation of T1 imaging as a tool for exploring cortical laminar composition. The full validation process is the accumulated result of multiple qualitative and quantitative assessments throughout the study: use of multiple datasets from different modalities (histology and neuroimaging) and species (human and macaque); visual assessment of the clusters as part of an initial global matching of regional patterns; assessment of results for spatially randomized datasets for comparison; and quantitative evaluations of the results, starting with hemispheric symmetry and culminating in correspondence with the granularity atlas. The correspondence between T1 layer clusters and regions with distinct cytoarchitectonic features shows that the T1 imaging framework enables cortical laminar composition analysis (Shamir et al. 2019, 2022; Shamir and Assaf 2021a, b).

Additional achievements of this study include demonstrating the adaptability and applicability of the BANKSY algorithm (Singhal et al. 2022) to neuroimaging data, as well as demonstrating the applicability of the framework in exploring the cytoarchitectonic features behind unique skillsets, such as musicality or athleticism. In the future, the methodology presented here can be applied to large groups of subjects not only to create a new and updated in vivo atlas of cytoarchitectonic features but also to further characterize subject-specific features associated with various abilities and skills.

## Data Availability

The histological datasets generated and/or analyzed during the current study are available in the BigBrain repository, https://bigbrainproject.org/. The thirty-subject (*N* = 30) average neuroimaging dataset generated and/or analyzed during the current study are available in Matlab format in a GitHub repository, https://github.com/ittais/Laminar_Connectivity. Per-subject datasets available from the corresponding author upon reasonable request. The three additional (*N* = 3) neuroimaging subjects datasets used and/or analyzed during the current study available from the corresponding author upon reasonable request. The macaque (*N* = 1) neuroimaging dataset used and/or analyzed during the current study available from the corresponding author on reasonable request.
